# Atmospheric and Fog Effects on Ultra-Wide Band Radar Operating at Extremely High Frequencies

**DOI:** 10.3390/s16050751

**Published:** 2016-05-23

**Authors:** Nezah Balal, Gad A. Pinhasi, Yosef Pinhasi

**Affiliations:** Faculty of Engineering, Ariel University, Ariel 40700, Israel; nezahb@ariel.ac.il (N.B.); gadip@ariel.ac.il (G.A.P.)

**Keywords:** extremely high frequencies, FMCW radar, atmosphere, millimeter waves, Tera-Hertz frequencies

## Abstract

The wide band at extremely high frequencies (EHF) above 30 GHz is applicable for high resolution directive radars, resolving the lack of free frequency bands within the lower part of the electromagnetic spectrum. Utilization of ultra-wideband signals in this EHF band is of interest, since it covers a relatively large spectrum, which is free of users, resulting in better resolution in both the longitudinal and transverse dimensions. Noting that frequencies in the millimeter band are subjected to high atmospheric attenuation and dispersion effects, a study of the degradation in the accuracy and resolution is presented. The fact that solid-state millimeter and sub-millimeter radiation sources are producing low power, the method of continuous-wave wideband frequency modulation becomes the natural technique for remote sensing and detection. Millimeter wave radars are used as complementary sensors for the detection of small radar cross-section objects under bad weather conditions, when small objects cannot be seen by optical cameras and infrared detectors. Theoretical analysis for the propagation of a wide “chirped” Frequency-Modulated Continuous-Wave (FMCW) radar signal in a dielectric medium is presented. It is shown that the frequency-dependent (complex) refractivity of the atmospheric medium causes distortions in the phase of the reflected signal, introducing noticeable errors in the longitudinal distance estimations, and at some frequencies may also degrade the resolution.

## 1. Introduction

Millimeter wave radar systems operating in multi-frequency or Frequency Modulated Continuous Wave (FMCW) mode were intensively studied over the recent years for a variety of applications such as collision avoidance in automobiles [1,2,3], remote sensing [4,5,6], concealed weapon detection for homeland security needs [7,8], helicopter and aircraft landing control and other related areas. The selection of operating frequencies in the millimeter wave regime and modulation techniques is an important issue in the design of active sensors and significantly depends on atmospheric propagation and penetration depth through various materials.

Broadband FMCW radars enable high resolution in range and depth perception and in the detection of objects with a small radar cross-section. The demand for broadband radar systems and the deficiency of wide frequency gaps within the lower band spectra necessitate the utilization of higher frequencies at millimeter wavelengths in the extremely high frequencies (EHF) range above 30 GHz.

The EHF band (30–300 GHz) covers a wide frequency range, which is relatively free of users. It offers many advantages for high resolution radars, enabling detection and imaging of concealed objects with a small radar cross-section. Among the practical advantages of using millimeter and sub-millimeter wavelength radar systems is the ability to employ smaller transmitting and receiving antennas. Millimeter wave radars also operate well under bad weather conditions such as fog and haze, when other sensors fail to detect objects, and thus serve as an alternative to infrared and visible light cameras. They are good candidates for landing assistance instrumentation measuring the height from the ground during dust and haze conditions.

**Figure 1:**
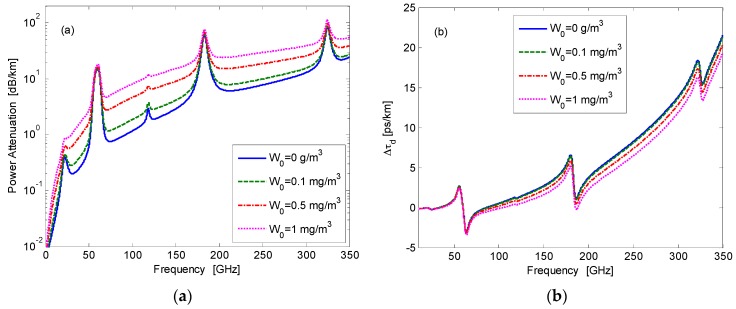
Atmospheric propagation factor in the EHF band: (**a**) attenuation coefficient 20log(e)⋅α(f) in (dB/km); (**b)** group delay increment $\Delta \tau_{d}=N\prime \left(f\right)\cdot d/c$ in (ps/km).

Some of the key challenges in the development of modern radar in the EHF band are the emerging effects during the propagation of electromagnetic radiation through the atmosphere. When millimeter wave radiation propagates in the atmospheric medium, it is subjected to selective molecular absorption [9,10,11,12,13,14]. Empirical and analytical models were suggested for evaluating millimeter and sub-millimeter wave propagation in the atmosphere. The transmission characteristics of the atmosphere in this band are shown in Figure 1 for different atmospheric water droplet concentrations, given by $W_{0}$ in (g/m^3^). The calculations were performed using the millimeter propagation model (MPM) developed by Liebe [15,16,17,18]. Millimeter wave signals propagating in the atmosphere suffer frequency-dependent absorptive and dispersive phenomena, causing distortions in amplitude and phase [19]. These effects should be considered in the realization of broadband radar systems [20,21,22]. While in millimeter wave communications atmospheric attenuation is the main cause of the reduction in the signal-to-noise ratio and thus leads to bit error rate growth, in FMCW radars, we show that atmospheric dispersion also plays an important role in the accuracy of distance measurements.

In the current paper, a general approach for studying ultra-wideband FMCW radars operating in the EHF band under conditions of fog and haze is developed. The study is aimed at pointing out the implications of how dispersive effects in the medium (and not only absorption) play a role on the performances of wideband FMCW radars. The numerical Millimeter-wave Propagation Model (MPM) [15,16] is employed for prediction of the atmospheric frequency response. The calculated propagation factor is used for the study of wide FMCW signal transmission through the atmosphere.

## 2. Propagation of a Linear FM Signal in a Dielectric Medium

We start with a general review of the description of electromagnetic radiation in the frequency domain. The fundamental principles of FMCW radars are also given here, mainly for convenience, defining the terms and notations used in the following discussion. The formulation we have developed is applied for the study of a linear FM (“chirp”) signal detected by a product detector after propagation and reflection from a target. We demonstrate the approach on a FMCW radar operating at millimeter and sub-millimeter wavelengths under fog conditions. We assume that the response of the components within the system is linear; this is a reasonable assumption as, though typical components have non-linear characteristics, it is possible in practice to use an equalizer to reduce the significance of this problem.

**Figure 2:**
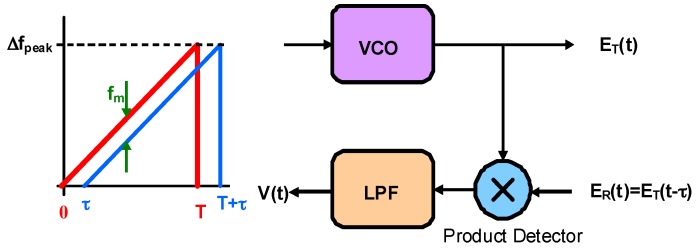
Block diagram of the linear FM radar.

In a FMCW radar (see Figure 2), a linear frequency-modulated signal (“chirp”) is transmitted toward the target. The instantaneous frequency of the transmission is changing linearly according to:

(1) $$f\left(t\right)=f_{c}\;+\frac{\Delta f}{T_{sweep}}t$$

where the carrier frequency is $f_{c}$ and the frequency span Δf divided by the sweep time $T_{sweep}$ is the compression ratio of the linear FM signal. The transmitted signal can be presented as a carrier wave at frequency $f_{c}$ modulated by a wideband signal:

(2) ET(t)=Re{AT(t)⋅ej2πfct}

Here $A_{T}\left(t\right)$ is a complex envelope, representing the baseband modulating signal. In the case of linear FM, the complex amplitude of the “chirp” is:

(3) $$A_{T}\left(t\right)=\exp\left(j\pi \frac{\Delta f}{T_{sweep}}t^{2}\right)$$

Fourier transform of the transmitted field Equation (2) yields:

(4) $$E_{T}(f)=\frac{1}{2}A_{T}\left(f-f_{c}\right)+\frac{1}{2}{A_{T}}^{*}\left[-\left(f+f_{c}\right)\right]$$

where $A_{T}\left(f\right)$ is the Fourier transform of the complex envelope $A_{T}\left(t\right)$ . The transmitted signal directed to the target located at a distance d is scattered back and arrives at the receiver after propagating a total distance of 2d in the dielectric medium, and the “phasor-like” field presentation in the positive frequency domain is given by:

(5) $${\tilde{E}}_{R}(f)=A_{T}\left(f-f_{c}\right)\cdot e^{-j2k_{z}\left(f\right)\;\cdot d}$$

where $k_{z}\left(f\right)$ is the complex propagation factor of the electromagnetic field in the medium. The Fourier transform of the field is:

(6) $$E\left(r,f\right)=\int_{-\infty }^{+\infty }E\left(r,t\right)\cdot e^{-j2\pi ft}dt$$

It is useful to present the time-dependent electric field in its analytic form, for which the Fourier transformation is given in positive frequencies [21]. The substitution of Equation (5) into Equation (6) results in the time-dependent signal obtained at the receiver input:

(7) ER(t)=Re{AR(t)⋅ej2πfct}

The complex envelope of the received signal is:

(8) $$A_{R}\left(t\right)=\int_{-\infty }^{+\infty }A_{T}\left(f\right)\cdot e^{-j2k_{z}\left(f+f_{c}\right)\;\cdot d}df$$

The local oscillator reference signal is formed from the same source as the transmitted FMCW signal and it is mixed with the delayed echoes reflected from the target. The mixed signal is filtered to remove higher-order harmonics. The resulting signal at the output of the filter is:

(9) $$\begin{array}{l} \tilde{V}\left(t\right)={A_{T}}^{*}\left(t\right)\cdot A_{R}\left(t\right)\\ ={A_{T}}^{*}\left(t\right)\cdot \int_{-\infty }^{+\infty }A_{T}\left(f\right)\cdot e^{-j2k_{z}\left(f+f_{c}\right)\cdot d}df \end{array}$$

The detected signal $\tilde{V}\left(t\right)$ is at baseband or intermediate frequencies. The distance to the target is measured via the instantaneous frequency resulting from the derivative:

(10) fIF(t)=−12πddtarctan  [Im{ V˜(t)}Re{ V˜(t)}]

If the signal is propagating in a vacuum, *i.e.*, when the index of refraction is a constant $n\left(f_{c}\;\right)=1\;$ , the propagation factor is k(f)z=2πf/c . As expected, the detected signal resulting from Equation (9) is a pure tone:

(11) $$\tilde{V}\left(t\right)=\exp\;\left(\begin{array}{l} -j2\pi \underset{f_{m}}{\underset{︸}{\frac{\Delta f}{T_{sweep}}\frac{2d}{c}}}\;t\\ +j\pi \frac{\Delta f}{T_{sweep}}{\left(\frac{2d}{c}\right)}^{2}-2\pi f_{c}\frac{2d}{c} \end{array}\right)$$

Here we define $\;f_{m}$ as the intermediate frequency (IF) obtained when the signal is propagating in vacuum. At a single frequency, $\;f_{m}$ is proportional to the distance to the target:

(12) $$d=\frac{T_{sweep}c}{2\Delta f}f_{m}$$

It is important to note that the longitudinal range resolution depends on the frequency resolution $\delta f_{m}$ :

(13) $$\delta d=\frac{T_{sweep}c}{2\Delta f}\delta \;f_{m}$$

Since the frequency resolution depends on the sweep duration $\delta \;f_{m}\approx 1/T_{sweep}$ , the error in the distance measurement is:

(14) $$\delta \;d=\frac{c}{2\Delta f}$$

The range resolution is improved as the radar bandwidth increases. A frequency span of Δf = 10 GHz results in a longitudinal resolution of $\delta \;d_{vacuum}\;=\;1.5\text{ cm}$ , which is not dependent on the distance (assuming a linear response of the medium). Very small objects can be detected while using an ultra-wideband signal. Since the frequency sweep in the FMCW signal is very large, one should consider the non-linearity that may emerge due to the inappropriate design of the radar RF chain [23]. A linear-phase frequency response is required to keep the linearity of the chirp along its whole temporal duration. Also, non-linear characteristics may cause a distortion in the modulated wave, resulting in further degradation in the sensitivity and thus an accuracy reduction in distance measurements. To overcome this issue, multi-stage amplification should be applied including limitation of the power gain to stay within the back-off regime of the amplifiers.

In order to focus the study only on the atmospheric propagation effects, a completely linear chirp is assumed to be transmitted. It is assumed that the FMCW signal is transmitted and received by a linear system in order to explore the impact of the atmospheric medium. Dispersive behavior of the atmospheric medium will cause deviations in the frequency calculated in Equation (10), and thus produce errors in the measured distance. We now demonstrate the effects of atmospheric fog on the resulting measured distance at millimeter wavelengths.

## 3. Millimeter Wave Propagation in the Atmosphere

The millimeter wave propagation model is based on a complex presentation of the refraction index:

(15) $$n\left(f\right)=1+N\left(f\right)\times 10^{-6}$$

The complex refractivity $N(f)=N_{0}+N\prime (f)-jN\prime \prime (f)$ is given in parts-per-million (ppm) [15,16]. The propagation factor $k_{z}\left(f\right)$ can be written in terms of the index of refraction:

(16) $$\begin{array}{l} k_{z}\left(f\right)=\frac{2\pi f}{c}n(f)=-j\;\underset{\alpha \left(f\right)}{\underset{︸}{\frac{2\pi f}{c}N\prime \prime \left(f\right)\times 10^{-6}}}\\ +\underset{\beta \left(f\right)}{\underset{︸}{\frac{2\pi f}{c}\left(1+N_{0}\times 10^{-6}\right)+\overset{\Delta \beta \left(f\right)}{\overset{︷}{\frac{2\pi f}{c}N\prime \left(f\right)\times 10^{-6}}}}} \end{array}$$

where c is the speed of light in a vacuum. The attenuation factor is:

(17) α(f)=−Im{kz(f)}=2π fcN″(f)×10−6

The wavenumber of the field is given by:

(18) β(f)=Re{kz(f)}=2πfc(1+N0×10−6)+2π fcN′(f)×10−6

The group delay at a distance d is defined via the derivative of the wavenumber:

(19) $$\begin{array}{l} \tau_{d}=\frac{d}{2\pi }\frac{d\beta }{df}\\ =\frac{d}{c}\left(1+N_{0}\times 10^{-6}\right)+\frac{d}{c}\left[N\prime \left(f\right)+f\frac{dN\prime }{df}\right]\times 10^{-6} \end{array}$$

Liebe’s millimeter propagation model (MPM) is used for calculation of the atmospheric frequency response under foggy conditions [15,16,17,18]. The results of this calculation are shown in Figure 1. Curves are drawn for several values of water droplet concentration. Absorption peaks are placed at 22 GHz and 183 GHz and 325 GHz, where the resonant absorption of water ( $H_{2}O$ ) occurs. Oxygen molecule ( $O_{2}$ ) absorptions are at 60 GHz and 119 GHz [24,25,26]. Atmospheric transmission windows are located between these frequencies, at 35 GHz (Ka-band), 94 GHz (W-band), 130 GHz and 220 GHz [12].

## 4. Numerical Results

Now we examine the absorptive and dispersive effects of fog on the propagation of an ultra-wideband ‘chirp’ transmitted at millimeter and sub-millimeter wavelengths and received by the FMCW detector. Note that both the attenuation coefficient α(f) and the wave number β(f) play a role in the wave propagation due to their non-uniform frequency response. The resulting detection leads to a deviation in the distance measurements. The MPM model also allows considerations of limited visibility due to fog and haze [16]. Fog and cloud conditions are characterized via the water droplet concentration W (in (g/m^3^). Figure 1 shows the effect of different values of droplet concentration on the attenuation and phase of a millimeter and THz wave.

**Figure 3:**
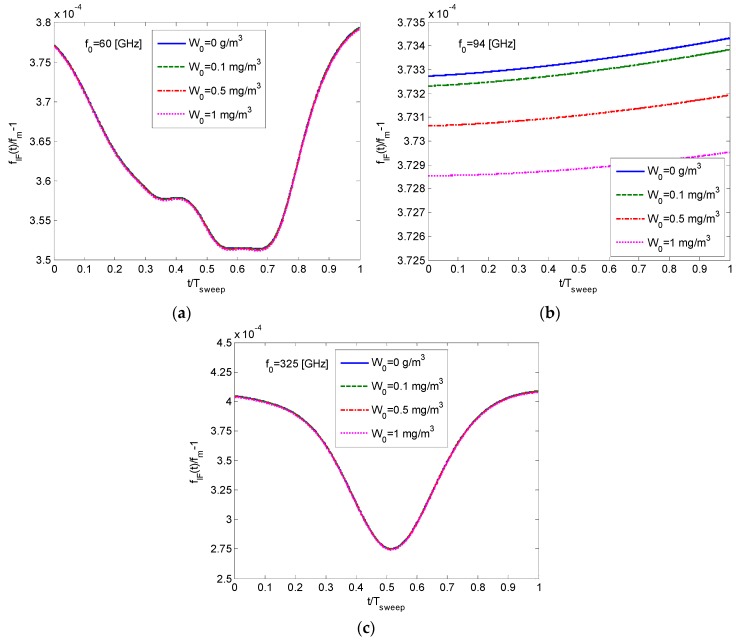
Variation of the instantaneous frequency $f_{IF}\left(t\right)/f_{m}-1$ obtained for different frequencies and fog conditions. (**a**) 60 GHz; (**b**) 94 GHz; (**c**) 325 GHz.

The intermediate frequency (IF) obtained at the output of the detector is given in Equation (10). It is proportional to the distance to the target, as can be seen in Equation (12). In the following, we consider a wideband FMCW millimeter wave radar transmitting a chirp with Δf = 10 GHz and $T_{sweep}\;=\;1\text{ ms}$ . A study of the resulting intermediate frequency is carried out for different frequency regimes centered at 60 GHz, 94 GHz, 120 GHz, and 325 GHz. For a given frequency sweep, the normalized detected IF frequency $f_{IF}\left(t\right)$ is shown in the graphs of Figure 3. The normalization is done by dividing the resulting frequency by $\;f_{m}$ , which is the IF expected frequency if the wave would have propagated in a vacuum.

Inspection of the graphs in Figure 3 reveals that $f_{IF}\left(t\right)>f_{m}$ during the whole sweep time. This means that there is an up-shift in the detected intermediate frequency; the resulting IF is higher than $f_{m}$ which is expected when the chirped signal is propagating in a vacuum (see Equation (11)).

Its average value is explained by the constant component $N_{0}$ in the atmospheric refractivity (see Equation (15)), causing an increase in the overall path delay. The frequency-dependent dispersive response N′(f) is the reason for the temporal variations in the intermediate frequency at the receiver output.

## 5. Distance Measurement Accuracy and Resolution

In the following we demonstrate that the deviation in the detected intermediate frequency causes errors in the absolute range estimation and may also lead to some degradation in the longitudinal resolution.

We previously showed that according to Equation (14), the longitudinal range resolution of $\delta \;d_{vacuum}=1.5\text{ cm}$ can be obtained by the wideband FMCW radar. This value is not affected by the distance to the target, nor by free space loss and attenuation of the wave while propagating in the atmosphere. Power loss causes a decrease in the signal-to-noise ratio, reducing the accuracy of the absolute distance measurement. We assume here that the received signal strength is well above the minimum detected signal and there is enough fade margin. This can be achieved by employing directive antennas, compensating for the low power [27,28].

It was shown in the previous section that in the dispersive atmosphere, the frequency $f_{IF}(t)$ of the detected signal is deviated from the expected $f_{m}$ , resulting in an error in the estimated range. Under foggy conditions, the distance to target is calculated by:

(20) $$d_{fog}\left(t\right)=\frac{T_{sweep}c}{2\Delta f}f_{IF}(t)$$

The difference between the measured value in foggy conditions and the real distance to the target (determined by $f_{m}$ ) is:

(21) $$\Delta d=d_{fog}\left(t\right)-d=\frac{T_{sweep}c}{2\Delta f}\left[f_{IF}(t)-f_{m}\right]$$

Using Equation (12) we obtain:

(22) $$\Delta d=\frac{f_{IF}(t)-f_{m}}{f_{m}}\cdot d$$

**Figure 4:**
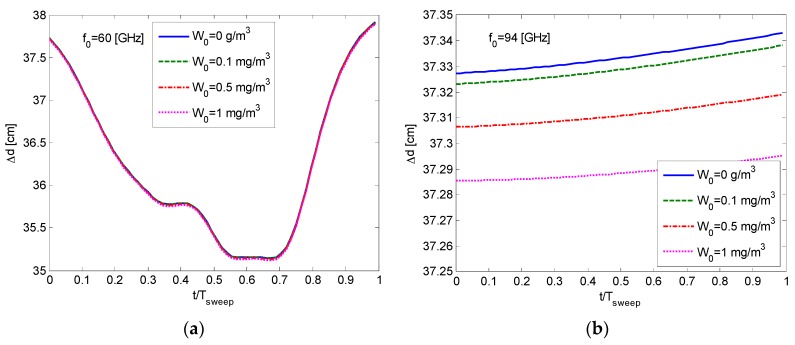

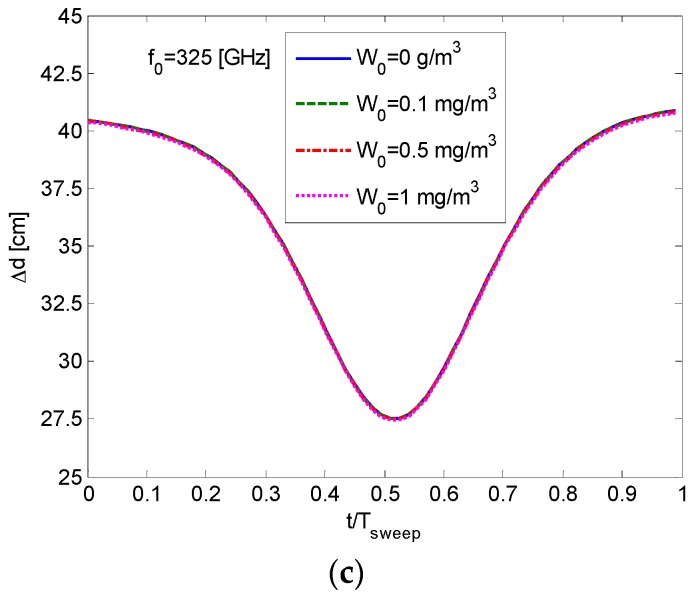
The range error in atmospheric foggy conditions. (**a**) 60 GHz; (**b**) 94 GHz; (**c**) 325 GHz.

Figure 4 illustrates the resulting error in the distance measurement for the discussed FMCW millimeter wave radar with Δf = 10 GHz and $T_{sweep}\;=\;1\text{ ms}$ while detecting an object at a distance of d = 1 km under foggy conditions. We choose to demonstrate the effect at frequencies of 94 GHz and 325 GHz, which are being used in many wideband millimeter and sub-millimeter radars.

The graphs of Figure 4 show that under foggy conditions, there is a systematic offset Δd in distance measurements, depending on the carrier frequency. This offset leads to an erroneous estimation $\bar{\Delta d}/d$ of the absolute distance to the target (here $\bar{\Delta d}$ is the average value of the offset from the real distance d ). The calculated range appears greater than the actual one.

Moreover, it can be seen that during the sweep time, the error deviation varies between a minimum value $\Delta d_{\min}$ to a maximum $\Delta d_{\max}$ , leading to a spread in the longitudinal point response. This instantaneous variation is due to the frequency-dependent dispersion component in Equation (15) and may cause a degradation $\left(\Delta d_{\max}-\Delta d_{\min}\right)/\delta d$ in resolution in some frequency bands. This error increases with the distance.

Table 1 presents calculations of the percentage errors in distance measurements $\bar{\Delta d}/d$ and resolution degradations $\left(\Delta d_{\max}-\Delta d_{\min}\right)/\delta d$ for several frequencies in the millimeter and sub-millimeter bands for a target 1 km away. Broadening the frequency sweep Δf (in our case up to 10 GHz) of the chirp, atmospheric conditions will not only affect the amplitude of the received signal (due to absorption) but also its phase, leading to a reduction in accuracy of distance estimations. The transmission of wide FMCW signals in atmospheric absorption resonances causes some degradation in resolution, as seen at 60 GHz, 183 GHz and 325 GHz. In imaging radar systems [27], the time-varying range offset would degrade the longitudinal resolution.

## 6. Conclusions

In this paper we developed a model to study the effect of fog on the distance accuracy measurements using an ultra-wideband FMCW radar operating in the millimeter and sub-millimeter wave regimes. The developed model can be employed to calculate the radar performance at any frequency from 10 GHz up to 1 THz. The study demonstrates the effects of dispersive attenuation and phase on a detected radar signal. It is found that loss and dispersion in the atmospheric medium cause a deviation in the frequency obtained at the output of the FMCW radar, leading to an erroneous estimation of the distance to the target. We found that foggy conditions significantly affect the accuracy of the ultra-wideband radar. If weather conditions are known (fog, mist, high humidity) or the transfer function of the medium is estimated, these effects can be overcome by an equalizer and adaptive system. ‘Side information’ about the channel behavior may be used for calibration of the system and compensating for the erroneous measurements. One should note that time-dependent variations in the atmospheric medium lead to temporal fading and turbulence scintillation phenomena. However, in this paper we assume that temporal changes in weather conditions are much slower than the travel time of the signal.

The obtained results are the physical bounds for accuracies in distance measurements as well as the resolution. Technical design issues, such as system non-linearities, can cause further degradation that should be considered. It is also interesting to note that in digital wireless communication wideband links operating in millimeter wavelengths, the dispersion of the atmospheric medium is expected to play an equivalent role. The magnitude and phase response of the atmosphere will affect the transfer of M-ary (multi) phase or frequency-shift keying signals, causing inter-symbol interference in the demodulated data.

## Figures and Tables

**Table 1:** Distance accuracy $\bar{\Delta d}/d$ and resolution degradation $\left(\Delta d_{\max}-\Delta d_{\min}\right)/\delta d$ when the target is at a distance of d = 1 km .

Frequency (GHz)	$\frac{\bar{\Delta d}}{d}$	$\frac{\Delta d_{\max}-\Delta d_{\min}}{\delta d}$
23	0.03715%	-
35	0.03725%	-
60	0.03630%	173%
77	0.03735%	-
94	0.037329%	-
120	0.03715%	-
183	0.03325%	700%
220	0.03785%	-
325	0.03425%	833%
